# Threat through the Screen? Association between Proximity and/or Watching Media Coverage of a Terrorist Attack and Health

**DOI:** 10.3390/ijerph20042876

**Published:** 2023-02-07

**Authors:** Roel Van Overmeire, Lise Eilin Stene, Marie Vandekerckhove, Stefaan Six, Reginald Deschepper, Johan Bilsen

**Affiliations:** 1 Mental Health and Wellbeing Research Group, Department of Public Health, Vrije Universiteit Brussel, 1090 Brussels, Belgium; 2 Norwegian Centre for Violence and Traumatic Stress Studies (NKVTS), 0484 Oslo, Norway; 3 Faculty of Psychology & Educational Science, Faculty of Medicine and Pharmaceutical Sciences, Vrije Universiteit Brussel (VUB), 1050 Brussels, Belgium; 4 Faculty of Arts and Philosophy, University of Ghent, 9000 Ghent, Belgium

**Keywords:** terrorism, mental health, media, somatic health, proximity

## Abstract

Introduction: After terrorist attacks, media coverage of the attacks is extensive. There are some indications that there is an association between watching the media coverage and certain health reactions, both mental and somatic. Most studies occur in the United States and often months after the initial attack. In the current study, we investigated the terrorist attacks in Belgium on 22 March 2016. Methods: An online cross-sectional survey was conducted one week after the attacks among the general population of Belgium. We measured hours of media watching of the terrorist attacks (hereafter media watching), adjusted scales of the Patient Health Questionaire-4 (PHQ-4) to measure mental symptoms and the Patient Health Questionaire-15 (PHQ-15) to measure somatic symptoms, proximity to Brussels (home, work and overall proximity) and background factors such as gender, age and level of education. Respondents were included if they answered the survey between 29 March 2016 and 5 April 2016. Results: A total of 2972 respondents were included. Overall, media watching was significantly associated with both mental symptoms (*p* < 0.001) and somatic symptoms (*p* < 0.001), while controlling for age, gender, level of education and proximity. Watching more than three hours of media was associated with more mental and somatic symptoms (*p* < 0.001). Compared to proximity, media watching was, in general, a better association. For geographical factors, watching more than three hours of media indicated equally high scores for mental symptoms and somatic symptoms as work proximity (*p* = 0.015) and overall proximity to the attacks (*p* = 0.024). Conclusion: Media-watching is associated with acute health reactions after terrorist attacks. However, the direction of the relationship is unclear, as it might also be that people with health issues seek out more media.

## 1. Introduction

After terrorist attacks, media will generally focus on these attacks in 24 h news cycles, bringing the latest news and rumors surrounding the attacks [1]. Research indicates that watching such terrorist attacks and their aftermath through the media is associated with health issues. Most of the studies investigating this were based on the attacks in the United States on 11 September 2001 (hereafter 9/11). One such study showed that post-traumatic stress reactions could be linked with seeing images of the attacks on the Twin Towers, as well as seeing people jump out of the towers [2]. Another study, in a representative sample in the U.S., showed that 22% of the population reported 9/11 to be their worst life event, even though they only viewed it through the media [3]. A study conducted 1–3 weeks after 9/11 showed that watching more than four hours of 9/11-related television, combined with cumulative acute stress, predicted both mental and physical health issues, two to three years later [4]. On the other hand, in a study of people directly exposed, media watching was not a good predictor of mental health issues [5]. Such results are also seen in one of the few European studies: after the Paris attacks on 13 November 2015, media watching of terrorist attacks was associated with post-traumatic stress reactions. However, this was also not present in those directly exposed to the attacks [6]. Other factors may explain the variability in the association. Higher age, lower socio-economic status, and female gender are associated with a higher chance of having mental health issues due to media watching [6].

However, there are a few gaps in prior studies. First, many studies are performed long after the initial event. For example, some are performed one to two months later [7], seven months later [6] to even 35 months later [8]. As these studies asked the respondent to recall months later how much media on a terrorist attack someone watched during the days after the attack, a recall bias is highly probable. For instance, someone with mental health problems following the attacks, might have a more vivid recollection of how much media they watched.

Second, while most studies of media watching and terrorism have concentrated on post-traumatic stress disorder, this has led to a gap in our knowledge of other mental health reactions. In fact, as of 2013, the new version of the DSM-V (diagnostic and statistical manual of mental disorders 5) removed media-exposure to violent events as a possible inclusion for post-traumatic stress disorder [9]. Considering the enormous number of studies showing depression and anxiety being linked with terrorist attacks, it is surprising that not more studies have looked at media watching and these mental health issues [10]. Especially when taking into account that general media studies showed that people with depression and anxiety disorder tend to use more media than healthy controls [11].

Third, there is a reason to suspect that media watching can also be linked with somatic reactions (see [4]). For example, some studies found that cardiovascular ailments might increase due to stress reactions upon seeing terrorist attacks [12,13]. Then again, other studies found no such reaction [14]. Thus, the relation between seeing terrorist attacks through media and certain somatic reactions remains unclear.

Fourth, it remains unclear in what way proximity plays a role in relation to media watching. We know that being directly exposed is a better predictor than media watching [5,6], but working or living close to the area of the attack remains understudied when combined with media watching.

Finally, as most studies were conducted in the U.S., and even in relation to 9/11, it remains difficult to generalize the association between media watching and mental health issues [6].

In the current study, we investigated media watching after the terrorist attacks in Belgium of 22 March 2016. On 22 March 2016 (hereafter 22 March 2016), two terrorist attacks occurred in Belgium. The first attack took place at the national airport, called Brussels Airport. Just before eight a.m., a suicide bomber near a coffee shop. Immediately after this, another suicide bomber struck near a check-in. A third man, who was supposed to do the same, fled the scene and was arrested weeks later. Approximately an hour after the airport attacks, a second attack took place while a metro was leaving the government area, involving a male suicide bomber. In total, 32 people were killed, in addition to the three suicide bombers, and hundreds were wounded [15]. The national threat level, indicating the chance of attacks on the nation’s soil, was increased to the maximum level, though it was lowered again by one level a couple of days later. Across the nation, people felt threatened by these attacks and feared further attacks [16]. Media outlets focused extensively on this terrorist attack, with many images of the massacre in the airport spreading, as well as videos of people escaping the metro station through the unlighted tunnels. Meanwhile, rumors spread around the internet of more threats, with bomb threats near the university in Brussels and even messages from gunmen on the roof of the airport.

Despite the extensive media coverage, there have been no studies on the association between media watching and health after the terrorist attacks in Belgium. It is important to have variation in studies on media watching, as media coverage might differ between countries. Furthermore, the current study is not hampered by possible recall bias, as it was conducted one week after the attacks in Belgium. Thus, in the present study, we investigated the relationship between media watching and health in Belgium, one to two weeks after the attacks.

We aimed to investigate:

1. The relation between media watching and mental health outcomes, namely anxiety and depressive symptoms.
2. The relation between media watching and physical health outcomes.
3. The role of proximity in relation to media watching in association with health outcomes.

## 2. Materials and Methods

### 2.1. Design

Similar to previous studies on this topic, we used a cross-sectional design [6,16]. Due to the short notice, being one week after the attacks, we used an online survey, as this was the quickest way to collect data. The survey was open to be completed one week after the attacks of 22 March 2016. Information about the survey was spread through the Belgian media (regional TV stations, radio and some widespread newspapers). The survey could be completed in either French, Dutch or English.

### 2.2. Population and Sample

Every person 18 years or older could complete the online survey. Respondents were included if they completed the survey between 29 March and 5 April 2016. Respondents were excluded if they had not completed the three key outcomes in full: media watching, PHQ-15 and PHQ-4.

### 2.3. Measures

Demographic information included age (continuous), gender and education level, where lower means “no diploma or no secondary school degree”, middle “secondary school degree” and higher means “university degree or higher”. Living situation was asked with the options “living alone”, “together with a partner”, “together with one or more persons who are not a partner” and “other”.

#### 2.3.1. Media-Watching

Similar to other studies e.g., [16], media watching was measured by asking how much media respondents had watched on average per day in the week of the attacks. The options were: less than an hour, 1–2 h, 2–3 h, more than 3 h. The type of media was not specified, so this could be a news report or social media, on television or on the internet. Throughout, this variable will be referred to as “media watching”, though it should be emphasized that this is the media watching of the terrorist attacks and not general media watching.

#### 2.3.2. Proximity

To estimate the proximity of people in relation to the attacks, the postcode of their home and the postcode of where they work/studied was asked. We recoded the postcode to “living in Brussels” and “Not living in Brussels”, and “working in Brussels” and “not working in Brussels”. Hereafter, these will be called “home proximity” and “work proximity”.

To include people in “work proximity”, we asked for their professional status. Respondents could indicate if they were students, wage-earners, independent, retired, disable/sick-leave or unemployed. For “work-proximity”, we looked at people who were either wage-earner, independent or students. It should be noted that a respondent could be, for example, “retired”, but also “independent”, as the question had multiple choice answers. Furthermore, a respondent could indicate to work in Brussels, but be unemployed. Thus, to realistically account for people working or studying in Brussels, all people who were retired, disable/sick-leave or unemployed were left out of the work-proximity sample.

After accounting for both home proximity and work proximity, we added an extra variable where we compared a group of people both working and living in Brussels with a group not working and living in Brussels. This variable was called “Overall proximity”.

#### 2.3.3. Health Indicators

PHQ-4 (Patient Health Questionnaire-4) was used to measure depressive symptoms and anxiety symptoms. We adjusted the scale to measure symptoms in the week of the attacks instead of over two weeks (*p* = 0.877). In total, there were four questions. Questions include “During the week of the terrorist attacks, how often have you been bothered by the following problems: feeling nervous, anxious or on edge?”, where respondents could answer from 0 (not at all) to 3 (nearly every day). The total range of the scale is from 0 to 12, with higher scores indicating more problems. The cutoffs are as follows: normal (0–2), mild (3–5), moderate (6–8) and severe (9–12) [17].

PHQ-15 (Patient Health Questionnaire-15) was used to measure somatic symptoms. As with PHQ-4, it was adjusted to measure the symptoms in the week of the attack (*p* = 0.836). It includes 15 questions, where respondents answered questions on what symptoms bothered them during the week of the attacks. Symptoms included stomach pain, headaches, dizziness, etc. Respondents could answer going from 1 (not bothered at all) to 3 (bothered a lot). The range of the scale is from 0 to 30, with higher scores indicating more problems. Cutoffs are at less than 5 (normal), 5 or more (mild), 10 or more (moderate) and 15 or more (severe) [18].

For both PHQ-4 and PHQ-15, we will mainly use the terms mental symptoms and somatic symptoms in the result section to indicate that these are not screenings of mental disorders and to avoid medicalizing the reactions.

### 2.4. Analysis

PHQ-4 and PHQ-15 scores were compared for home proximity and work proximity through independent t-tests. Through a chi-square test, we compared, on the one hand, proximity and the cutoffs of PHQ-4 and PHQ-15, and, on the other hand, media watching. Here, media watching was recoded to compare three hours of watching or less with three hours and more. This was also conducted for education level and media watching. The living situation was recoded to “Alone” and “Together” and compared to media watching. To check for interaction effects, we have employed a two-way ANOVA, with first, media watching and proximity as factors, and PHQ-4 and PHQ-15 as outcomes.

Finally, based on previous studies, hierarchical linear regressions were used where the outcome was either PHQ-4 or PHQ-15 [16]. Two levels were used each time, with on the first level gender, age, education level and proximity, and on the second, media watching. Education level was recoded so that “lower-middle educated” was the reference category. We have used three different variations each time for both the PHQ-4 and PHQ-15 as outcomes, where proximity was once work proximity, once home proximity and finally overall proximity. Thus, in total, there were six models. Each time, B-values, standard deviations, p-values and R^2^-values were reported, where R^2^ values were always adjusted R^2^ values.

### 2.5. Ethics

The Medical Ethics Committee of UZ Brussels/VUB approved this study (B.U.N. 143.201.526.618).

## 3. Results

### 3.1. Characteristics of Sample

In total, 3635 people answered the survey. However, 663 were excluded in total, of which 265 were due to missing values for our three main outcomes and 398 were due to answering the survey after the 5th of April. After exclusion, 2972 respondents were included in the study.

The mean age of respondents was 41.8 (±13.969), with a range of 18–86. In terms of gender, 2077 (69.9%) were female and 895 (30.1%) were male. 255 (8.6%) were lower educated, while 1811 (60.9%) were higher educated, with 906 (30.5%) remaining as middle educated. For professional status, 1948 were wage-earners (65.5%), 326 were independent (11%), 361 students (12.1%). Furthermore, 120 people were unemployed (4%), 236 were retired (7.9%) and 112 on sick leave (3.8%). After exclusion, a total of 2507 (84.4%) were in the work category. Ten did not complete the proximity question, leaving 2497 (84%) in the work-proximity group. Of these, 1231 worked in Brussels (49.3%) and 1266 did not work in Brussels (50.7%). For home proximity, 793 lived in Brussels (26.7%), while 1231 (41.4%) worked in Brussels. There were eight missing (see Table 1).

The overlap between the two proximities shows that 640 people who lived in Brussels also worked there (*p* < 0.001), while 1200 did not work or live in Brussels. This formed the sample for overall proximity (N = 1840) (see Table 2).

Overall, 16.2% of respondents had severe acute mental health reactions, while 6.3% had severe acute somatic reactions. For both mental and somatic reactions, the majority had either normal reactions or mild reactions. Mental symptoms had an average score of 4.512 (±3.5144), while for somatic symptoms this is 5.54 (±4.937).

### 3.2. Media-Watching

In total, 1185 respondents (39.9%) watched more than three hours of media on the terrorist attacks during the week of the attacks. There was a significant difference in the amount of media watched for education levels (*p* = 0.003) and for living situations (*p* = 0.010) (see Table 3).

### 3.3. Proximity to Attacks

Mental symptom values differed significantly for home proximity (*p* < 0.001). Region Brussels had a mean of 5.1740 (±3.5869), while people living outside of Brussels had 4.2736 (±3.4518). For those working in Brussels, the mean was 4.7929 (±3.4406), while those not working in Brussels had a mean of 4.1927 (±3.4247) (*p* < 0.001).

Somatic symptom averages were also significantly higher (*p* = 0.002) for people living in Brussels, with an average of 6.0 (4.881), compared to not living in Brussels, 5.37 (±4.952). For working in Brussels, this was also the case (*p* = 0.035). Mean values for working in Brussels were 5.70 (±4.830) and not working in Brussels were 5.29 (±4.825).

For those with an overall close proximity, similar results were found. Mental symptom averages for Brussels were 5.1813 (±3.5632), not Brussels 4.1733 (±3.4207) (*p* < 0.001), while for PHQ-15 these were 6.01 (±4.87) and not Brussels 5.29 (4.869) (*p* = 0.003) (see Table 4).

### 3.4. Proximity and Media-Watching

Media-watching and home proximity had a significant difference (*p* < 0.001), while working in Brussels and media-watching did not (*p* = 0.104). Overall proximity was significant (*s* = 0.003) (see Table 5).

**Figure 1:**
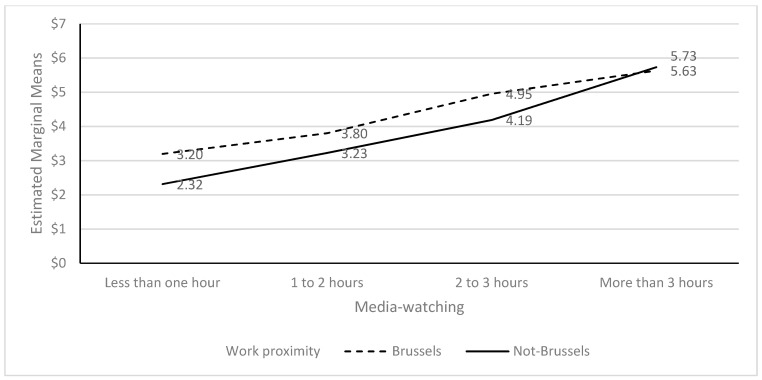
PHQ-4 as an outcome of the interaction between media watching and work proximity.

Two-way ANOVA’s showed a lack of an interaction effect for home proximity and media-watching for mental symptoms (F = 2.273; *p* = 0.078). The media (F = 74.228; *p* < 0.001) and home proximity (F = 15.793; *p* < 0.001) were both significant. However, there was an interaction effect for work proximity and media-watching on mental symptoms (F = 3.483; *p* = 0.015). While there was a difference between watching three hours or less of media and proximity, with being closer being associated with a higher mental symptoms score, this reversed for watching more than three hours. Both media (F = 93.497; *p* < 0.001) and work proximity (F = 13.387; *p* < 0.001) were significant (see Figure 1).

**Figure 2:**
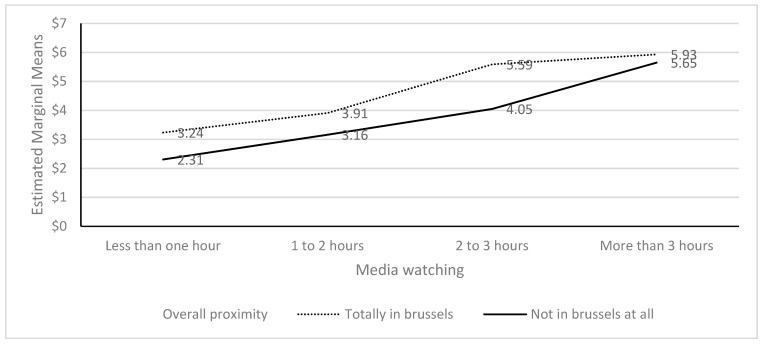
PHQ-4 as outcome for media watching and overall proximity.

Overall, proximity had a significant interaction with media watching for mental symptoms (F = 3.145; *p* = 0.024). While the lines did not intersect, the means for more than 3 h of media watching and proximity with regard to mental symptoms are almost the same, indicating that the difference between living and working in Brussels was not different in terms of mental symptoms compared to watching more than three hours of media on the attacks. The variables media watching (*p* < 0.001) and proximity (*p* < 0.001) were both significant predictors in the analyses (see Figure 2).

For somatic symptoms, there was no interaction effect for home proximity and media watching (F = 1.396; *p* = 0.242). Home proximity was not significant (F = 2.640; *p* = 0.104), and media watching was (F = 46.918; *p* < 0.001). For neither work proximity nor media-watching was there an interaction effect (F = 1.369; *p* = 0.250). Here, work proximity was not significant (F = 2.935; *p* = 0.087), while media watching was (F = 63.310; *p* < 0.001). Overall, proximity had no interaction effect with media (F = 1.609; *p* = 0.185). Media watching was significant (F = 35.615; *p* < 0.001) and proximity as well (F = 3.874; *p* = 0.049).

Finally, we performed five hierarchical linear regressions. For mental symptoms, we found that for every model, media-watching and proximity were both significant, though in all models, the R^2^ at least doubled after inclusion of media-watching. For somatic symptoms, work proximity was initially significant (*p* = 0.017), but after including of media watching (*p* < 0.001), the p-value increased to 0.110. Age was never significant for somatic symptoms. Here too, media watching each time doubled the R^2^ score. Gender was significant for every step in every model, both mental symptoms and somatic symptoms, each time with a *p*-value below 0.001, with males always having fewer mental or somatic symptoms compared to the reference group of females. Similarly, being highly educated was associated with fewer mental and somatic symptoms (see Table 6).

## 4. Discussion

In the present study, we looked at the association between watching media coverage on terrorist attacks and proximity to the attacks 1–2 weeks after the terrorist attacks in Belgium of 22/03/2016. Our results suggest that, although proximity was associated in an important way with mental and somatic symptoms, media watching had a seemingly stronger association, especially when watching more than three hours of media about terrorist attacks. However, it should also be noted that all health reactions were on average higher in Brussels than outside of Brussels, indicating again the role of proximity. Furthermore, in addition to media watching and proximity, factors such as age, gender and education level were also important. In general, the severity of mental and somatic symptoms was quite low. For mental health, 34.5% had either moderate or severe symptoms, while for somatic health, this was 19.4%. On average, the group watching more than three hours of media had scores bordering on mild mental and somatic symptoms.

In general, the associations we found were similar to those identified in prior studies. For example, studies after the Boston Marathon bombing and the Paris-attacks showed that more time spent media watching was associated with higher stress scores [6,19]. When looking at media watching, it seemed that watching more than three hours was associated with worse mental and physical health. For example, 16.2% of those watching for more than three hours of media had severe acute mental health symptoms. However, an important nuance is that the percentages were also relatively high for mental health symptoms for those watching less than three hours of media: 10.2%. Furthermore, 35% of those who watched more than three hours reported no mental health reactions. It should thus be remembered that when stating that watching too much media is associated with health reactions, we are discussing a relatively small group in our study.

Yet, then again, media watching does seem to fit better in association with health symptoms than proximity. In fact, proximity did not even play a role in predicting somatic reactions compared to media watching for work proximity and somatic reactions. In general, there were less severe somatic symptoms than those of mental health. That might indicate that the “threshold” to get such symptoms is higher. It is also possible that those with higher levels of somatic symptoms were more likely to watch media.

Another aspect might be that people who work are generally healthier than people who do not work; we excluded unemployed and retired people for work proximity. Hence, we cannot ascertain whether the higher symptom levels among those with high media watching were actually due to the media exposure to the attacks. Furthermore, the working sample did not include those out of work. Therefore, it is possible that there were overall lower levels of somatic symptoms in this sample than when we also included those who were out of work in the analysis of home proximity. Keeping that in mind, having one’s work in Brussels might also feel less threatening than living in Brussels. Living in Brussels may mean taking public transport more often, and the metro line that was bombed was an important metro line, going to the central station and to a university. Furthermore, people who worked in an area close to the attacks probably did not go to work during the week of the attacks due to the threat. For students, many stayed home during that week because reaching the university was difficult.

Surprisingly, for somatic reactions, age was never a significant predictor. Why that might be is unclear, though we would point out that even for mental health reactions, age had a weak association, albeit a significant one. The very low B-values indicated that age, in general, does not play a role in such reactions. That is quite similar to the study on media watching and the Paris attacks [6], while different from a study in the U.S. [4].

The direction of the relationship between symptoms and media watching is unclear. For example, it might be that people with mental health issues seek out more media about terrorist attacks. After all, non-terrorist media studies have shown that people with depression and anxiety disorder tend to use more media than healthy controls [11]. Explaining the direction is impossible with our cross-sectional design.

However, context is important to give insight into what might be more plausible. The attacks in Belgium were one in a series of attacks in Europe. Just months earlier, on 13/11/2015, France was attacked by terrorists whose base of operations was located in Belgium. In fact, just before the Belgium attacks, one of the lead terrorists of the France attacks had been arrested. Thus, it might be that there was already quite some distress among some people of the population, which led them to view more of the terrorist attacks in Belgium than the average person. That would support the findings in other studies, where media-watching and mental distress are in a sort of vicious cycle, each strengthening the other, and cumulatively building up [20]. A study on the media watching of 9/11 by Oklahoma City bombing survivors showed that there is reason to suspect that cumulative exposure to media and stress can be important in explaining the association [21].

However, would we not then expect the relationship between media watching and health reactions to be stronger? The Belgian attacks occurred after the media-covered attacks on Charlie Hebdo and the Paris attacks, in a climate of regular attacks. Yet, the relationship between media and health is quite mild, all things considered.

It might also be that people in Belgium had gotten used to the threat of terrorism. While there is hardly evidence of the public health impact of the Paris attacks, the number of suicides increased during the two-week lockdown period after the Paris attacks in Belgium, while there was no increase after the Belgian attacks [22]. It is not unthinkable that people in Belgium were “expecting” an attack sooner or later. Thus, perhaps those affected by the long-term threat also viewed more media during the Brussels attacks.

Comparing our results to the studies from the U.S. is difficult, as such studies take place in a different media landscape, e.g., Belgium has no 24/24 h news channels. Thus, we would need more European research on terrorism and media watching. If media plays an important role, it is necessary to look at the role of different media cultures. While certainly there is also a difference in media in Europe itself, the point is that the focus at the moment of media watching of terrorist attacks is primarily an American study field.

As mentioned, we have no indication of the long-term problems that might be related to media watching in Belgium. A study conducted 2.5 years after the attacks in Belgium showed that a sample in Brussels did show slightly fewer mental health issues than in the current study, but most importantly, it showed a significant association between mental health, the perception of terrorist threat and types of avoiding behavior: people avoided certain places associated with the attacks [23]. That cannot, however, not be linked to the media, as at that point, there was hardly any coverage of the attacks in Belgium. The attacks themselves probably left a mark on the people living in Brussels, affecting their behavior, and indicating the role of proximity after the media stopped reporting on an attack.

It may, however, be that this “mark” was created by the extensive media coverage at the moment of the attacks. Another interpretation in relation to the current study might be that people who feel distressed continue to fear future terrorist attacks, even after the media has stopped reporting on them. It might, therefore, be that these people are also the ones who will view a lot of media when a new terrorist attack occurs. In short, it remains unclear, though it might be reasonably expected that the association between media watching of terrorist attacks and health cannot be solely explained by one single attack [20].

Our study showed that media watching could be associated with health reactions during the week after an attack. Whether these symptoms will last is unclear. However, there is no reason to recommend balancing sensationalistic aspects of media coverage [20]. Considering the number of ways of accessing media (e.g., television, social media, the internet and newspapers), it seems more opportune to invest in public health promotion so that the general population is informed of the possible (short-term) adverse problems associated with high media consumption. Aside from such actions, we should trust the critical thinking of the general population to handle free and uncensored media [24].

This study is limited in several ways. First, it was an online survey with convenience sampling, and it is unlikely that this is a representative sample of the population. Our sample differs from several indicators of the population of Belgium. In our sample, 4% were unemployed, while during this period this was 7.6% in Belgium overall. For gender, in the general population, the difference is more toward 51% female, while in our sample, 69.9% were female [25]. Second, in measuring so soon after the attacks, we measured what were probably mainly normal reactions. It is unclear how many of these respondents developed long-term problems. Their reactions in this study should therefore not be medicalized. Third, it is impossible to make a causal connection: people with more emotional reactions might be more inclined to see more about the attacks. Fourth, we did not investigate what type of media people watched—it might be that social media is more harmful than regular news channels, for example.

The study has several strengths. The greatest strength is the lack of temporal lag in the data collection. While most media-watching studies in terrorism research take place a long time after the attack, potentially creating recall bias, this study sample completed the survey one to two weeks after the attacks, limiting the potential recall bias. Second, this study also took into account somatic reactions. This shows the diversity of symptoms associated with such attacks. Third, to our knowledge, this is the first study to look at the relationship between media watching and health after terrorist attacks in Belgium.

## 5. Conclusions

Media-watching of terrorist attacks and proximity to the attacks were associated with health. This study indicated that viewing more than three hours of media each day in the week of an attack can be associated with more mental and somatic symptoms. 

## Figures and Tables

**Table 1:** Characteristics sample.

	N	%
Gender		
Male	895	30.1
Female	2077	69.9
Education level		
Lower	255	8.6
Middle	906	30.5
Higher	1811	60.9
Professional status		
Wage earners	1948	65.5
Independent	326	11
Students	361	12.1
Unemployed	129	4
Retired	236	7.9
Sick leave	112	3.8
Living situation		
Alone	603	20.3
Together	2362	79.7
Home proximity		
Brussels	793	26.8
Not Brussels	2171	73.2
Work proximity		
Brussels	1231	49.3
Not Brussels	1266	50.7
Overall proximity *		
Brussels	640	34.8
Not Brussels	1200	65.2
Mental symptoms (PHQ-4)		
Normal	1040	35
Mild	907	30.5
Moderate	544	18.3
Severe	481	16.2
Somatic symptoms (PHQ-15)		
Normal	1500	50.5
Mild	895	30.1
Moderate	390	13.1
Severe	187	6.3

* Overall number lower due to overlap.

**Table 2:** Overlap proximities.

	Working in Brussels	%	Not Working in Brussels	%
Living in Brussels	640	52.1	65	5.1
Not living in Brussels	588	47.9	1200	94.4

4 missing.

**Table 3:** Media watching, living conditions and education level.

	Three Hours or Less Watching	%	More than Three Hours Watching	%	*p*-Value
Alone *	335	18.8	268	22.7	0.01
Together	1149	81.2	913	77.3	
Lower education level	129	7.2	126	10.6	0.003
Middle education level	544	30.4	362	30.5	
Higher education level	1114	62.3	697	58.8	
PHQ-4					<0.001
Normal	774	43.3	266	35	
Mild	577	31.2	350	30.5	
Moderate	274	15.3	270	18.3	
Severe	182	10.2	299	16.2	
PHQ-15					<0.001
Normal	1059	59.3	441	37.2	
Mild	486	27.2	409	34.5	
Moderate	175	9.8	215	18.1	
Severe	67	3.7	120	10.1	

* 7 missings for living situation.

**Table 4:** Averages of media watching and proximity, with health as outcome.

	PHQ-4	SD	*p*-Value	PHQ-15	SD	*p*-Value
Three hours or less watching	3.7448	3.1925	<0.001	4.61	4.434	<0.001
More than three hours watching	5.692	3.6519		6.95	5.313	
Living in Brussels	5.1740	3.5869	<0.001	6.0	4.881	0.002
Not living in Brussels	4.2736	3.4518		5.37	4.952	
Working in Brussels	4.7929	3.4406	<0.001	5.70	4.830	0.035
Not working in Brussels	4.1927	3.4247		5.29	4.825	
Overall in Brussels	5.1813	3.5632	<0.001	6.01	4.87	0.003
Overall not in Brussels	4.1733	3.4207		5.29	4.869	

**Table 5:** Overlapping media watching and proximity (column percentages).

	Three Hours or Less Watching	%	More than Three Hours Watching	%	*p*-Value
Living in Brussels	423	23.7	370	31.4	<0.001
Not living in Brussels	1361	76.3	810	68.6	
Total	1784	100	1180	100	
Working in Brussels	732	48	499	51.3	0.104
Not working in Brussels	793	52	473	48.7	
Total	1525	100	972	100	
Overall in Brussels	359	32.1	281	39	0.003
Overall not in Brussels	761	67.9	439	61	
Total	1120	100	720	100	

Totals do not add up to 2972 for home proximity, due to 8 missing.

**Table 6:** Regression analyses.

	B	SD	*p*	R^2^
PHQ-4				
Step 1			<0.001	0.058
Constant	8.345	0.367	<0.001	
Age	0.005	0.005	0.311	
Gender (female ref. cat)	−1.520	0.137	<0.001	
Education level (lower-middle ref. cat)	−0.595	0.131	<0.001	
Home proximity (Brussels. ref. cat)	−0.971	0.144	<0.001	
Step 2			<0.001	0.139
Constant	4.675	0.414	<0.001	
Age	0.007	0.004	0.091	
Gender (female ref. cat)	−1.339	0.132	<0.001	
Education level (lower-middle ref. cat)	−0.529	0.125	<0.001	
Home proximity (Brussels. ref. cat)	−0.750	0.138	<0.001	
Media-watching	0.986	0.059	<0.001	
PHQ-4				
Step 1			<0.001	0.049
Constant	7.105	0.369	<0.001	
Age	0.013	0.006	0.015	
Gender (female ref. cat)	−1.453	0.149	<0.001	
Education level (lower-middle ref. cat)	−0.472	0.142	<0.001	
Work proximity (Brussels. ref. cat)	−0.641	0.137	<0.001	
Step 2			<0.001	0.132
Constant	3.595	0.419	<0.001	
Age	0.018	0.005	<0.001	
Gender (female ref. cat)	−1.282	0.143	<0.001	
Education level (lower-middle ref. cat)	−0.445	0.136	0.001	
Work proximity (Brussels. ref. cat)	−0.502	0.131	<0.001	
Media-watching	0.985	0.064	<0.001	
PHQ-4				
Step 1			<0.001	0.065
Constant	8.044	0.46	<0.001	
Age	0.017	0.007	0.009	
Gender (female ref. cat)	−1.403	0.174	<0.001	
Education level (lower-middle ref. cat)	−0.780	0.167	<0.001	
Overall proximity (Brussels. ref. cat)	−0.574	0.085	<0.001	
Step 2			<0.001	0.16
Constant	4.14	0.512	<0.001	
Age	0.022	0.006	<0.001	
Gender (female ref. cat)	−1.255	0.165	<0.001	
Education level (lower-middle ref. cat)	−0.737	0.158	<0.001	
Overall proximity (Brussels. ref. cat)	−0.454	0.081	<0.001	
Media-watching	1.065	0.074	<0.001	
PHQ-15				
Step 1			<0.001	0.067
Constant	10.941	0.514	<0.001	
Age	−0.008	0.006	0.193	
Gender (female ref. cat)	−2.545	0.192	<0.001	
Education level (lower-middle ref. cat)	−0.900	0.183	<0.001	
Home proximity (Brussels. ref. cat)	−0.691	0.201	<0.001	
Step 2			<0.001	0.118
Constant	6.82	0.589	<0.001	
Age	−0.005	0.006	0.401	
Gender (female ref. cat)	−2.342	0.188	<0.001	
Education level (lower-middle ref. cat)	−0.825	0.178	<0.001	
Home proximity (Brussels. ref. cat)	−0.442	0.197	0.025	
Media-watching	1.108	0.084	<0.001	
PHQ-15				
Step 1			<0.001	0.059
Constant	9.77	0.514	<0.001	
Age	0.001	0.008	0.855	
Gender (female ref. cat)	−2.448	0.207	<0.001	
Education level (lower-middle ref. cat)	−0.776	0.198	<0.001	
Work proximity (Brussels. ref. cat)	−0.454	0.191	0.017	
Step 2			<0.001	0.114
Constant	5.785	0.593	<0.001	
Age	0.007	0.007	0.37	
Gender (female ref. cat)	−2.254	0.202	<0.001	
Education level (lower-middle ref. cat)	−0.745	0.192	<0.001	
Work proximity (Brussels. ref. cat)	−0.297	0.185	0.11	
Media-watching	1.118	0.09	<0.001	
PHQ-15				
Step 1			<0.001	0.057
Constant	10.459	0.642	<0.001	
Age	0.001	0.009	0.877	
Gender (female ref. cat)	−2.238	0.243	<0.001	
Education level (lower-middle ref. cat)	−1.051	0.233	<0.001	
Overall proximity (Brussels. ref. cat)	−0.433	0.119	<0.001	
Step 2			<0.001	0.117
Constant	6.174	0.731	<0.001	
Age	0.006	0.009	0.468	
Gender (female ref. cat)	−2.076	0.236	<0.001	
Education level (lower-middle ref. cat)	−1.004	0.226	<0.001	
Overall proximity (Brussels. ref. cat)	−0.301	0.116	0.009	
Media-watching	1.169	0.105	<0.001	

For proximity variables, “in Brussels” is the reference category. For gender, female is the reference category. For education level, lower-middle educated is the reference category.

## Data Availability

Data cannot be made available due to privacy concerns and as mandated by the Medical Ethics Committee of UZ Brussels/VUB.
